# Challenges in coupling atmospheric electricity with biological systems

**DOI:** 10.1007/s00484-020-01960-7

**Published:** 2020-07-14

**Authors:** Ellard R. Hunting, James Matthews, Pablo Fernández de Arróyabe Hernáez, Sam J. England, Konstantinos Kourtidis, Kuang Koh, Keri Nicoll, R. Giles Harrison, Konstantine Manser, Colin Price, Snezana Dragovic, Michal Cifra, Anna Odzimek, Daniel Robert

**Affiliations:** 1 School of Biological Sciences, University of Bristol, Bristol, UK; 2 School of Chemistry, University of Bristol, Bristol, UK; 3 Geography and Planning Department, Universidad de Cantabria, Santander, Spain; 4 Department of Environmental Engineering, Demokritus University of Thrace, Xanthi, Greece; 5 ISLP Xanthi Branch, ENTA Unit, ATHENA Research and Innovation Center, Xanthi, Greece; 6 Department of Electronic and Electrical Engineering, University of Bath, Bath, UK; 7 Department of Meteorology, University of Reading, Reading, UK; 8 Department of Geophysics. Porter School of the Environment and Earth Sciences, Tel Aviv University, Tel Aviv, Israel; 9 Vinča Institute of Nuclear Sciences, University of Belgrade, Belgrade, Serbia; 10 Institute of Photonics and Electronics, Czech Academy of Sciences, Prague, Czechia; 11 Institute of Geophysics, Polish Academy of Sciences, Warsaw, Poland

**Keywords:** Aerosols, Biometeorology, Ecosystem connectivity, Electromagnetics, Electroreception, Electrostatics, Ions, Lightning, Potential gradient, Radionuclides, Thunderstorm

## Abstract

The atmosphere is host to a complex electric environment, ranging from a global electric circuit generating fluctuating atmospheric electric fields to local lightning strikes and ions. While research on interactions of organisms with their electrical environment is deeply rooted in the aquatic environment, it has hitherto been confined to interactions with local electrical phenomena and organismal perception of electric fields. However, there is emerging evidence of coupling between large- and small-scale atmospheric electrical phenomena and various biological processes in terrestrial environments that even appear to be tied to continental waters. Here, we synthesize our current understanding of this connectivity, discussing how atmospheric electricity can affect various levels of biological organization across multiple ecosystems. We identify opportunities for research, highlighting its complexity and interdisciplinary nature and draw attention to both conceptual and technical challenges lying ahead of our future understanding of the relationship between atmospheric electricity and the organization and functioning of biological systems.

## Introduction

The Earth’s atmosphere is a complex physical environment that makes up an intrinsic component of our living environment. For decades, interactions between organisms (animals, plants, bacteria, fungi, archaea, and human beings) and their geophysical and geochemical environment have been a central avenue of empirical research (Halberg 1963). Despite these efforts, biophysical mechanisms underpinning interactions between many atmospheric variables and biological systems remain poorly understood. Thus far, the complexity and diversity of the physical processes operating simultaneously over wide spatio-temporal scales have hampered our understanding whether and how atmospheric physical processes—and their dynamics—can be related to multiple levels of biological organization ranging from molecular dynamics to the functioning of ecosystems.

The atmosphere is host to various sources of electrical variations, spanning spatial dimensions, and electric currents that range from the production of single electrons and ions to the ~ 1000 A global electric circuit of planetary scale (Rycroft et al. 2008). While interactions between organisms and their electrical environment have been mostly studied in the aquatic, electrically rather conductive, environment (Bullock et al. 2006; Crampton 2019), comparatively very little is known about how atmospheric electrical phenomena are tied to biology. However, emerging evidence points to atmospheric electricity interacting with various organisms over various levels of biological organization (e.g., ions, molecules, cells, and organisms; e.g., Morley and Robert 2018; Hunting et al. 2019). As evidence is beginning to highlight the responses of biological systems to known drivers of variations in atmospheric electricity, here we aim to offer several vantage points, synthesizing current understanding of atmospheric electrical phenomena and their interplay with various levels of biological organization. By briefly highlighting some of the prominent historical and contemporary examples, we hope to inspire further forays by other researchers in this fascinating field of interdisciplinary research. To this end, conceptual and technical challenges are identified, providing a platform for further discussions, collaborations, and opportunities for progress and innovation at the interface between meteorology, atmospheric physics, and chemistry, as well as biological and medical sciences.

## The atmospheric electrical environment

Various sources of electricity are present in the atmosphere, ranging from global electromagnetic fields and electrostatic fields to more local phenomena such as lightning and ions. Each of these electric phenomena have different degrees of pervasiveness and variability, and potential interactions with biology.

Electromagnetic fields are a ubiquitous physical aspect of the Earth’s atmosphere that historically received scientific attention, especially with respect to its relevance for biology (e.g., Palmer et al. 2006). Electromagnetic fields are composed of electric and magnetic fields of force, generated by natural phenomena or by humans with the use of electrical appliances (e.g., mobile phones, power lines and computers). Electromagnetic fields existing in nature and produced artificially exhibit a wide spectrum of frequencies, ranging from static and quasi-static range (< 3 Hz) to extremely high frequencies (300 GHz) in the microwave range of wavelengths (Mikolajczyk 1990; Saliev et al. 2019). The most well-known natural phenomenon is the static magnetic field of the Earth, putatively generated by electric currents in the melted iron core of the Earth’s core (Kuang and Bloxham 1997). The shape of the Earth’s magnetic field can be approximated by a magnetic dipole, but there may be notable local deviations in which the strength and the actual shape fluctuate on time scales of milliseconds and hours (Hayakawa et al. 2004) to millions of years (McElhinny and McFadden 1998). These natural atmospheric (and cosmic) electromagnetic fields are also an important driver of Earth currents (or telluric currents), and their dynamics, in both soil and water (for review see: Lanzerotti and Gregori 1986; Helman 2013).

**Fig. 1:**
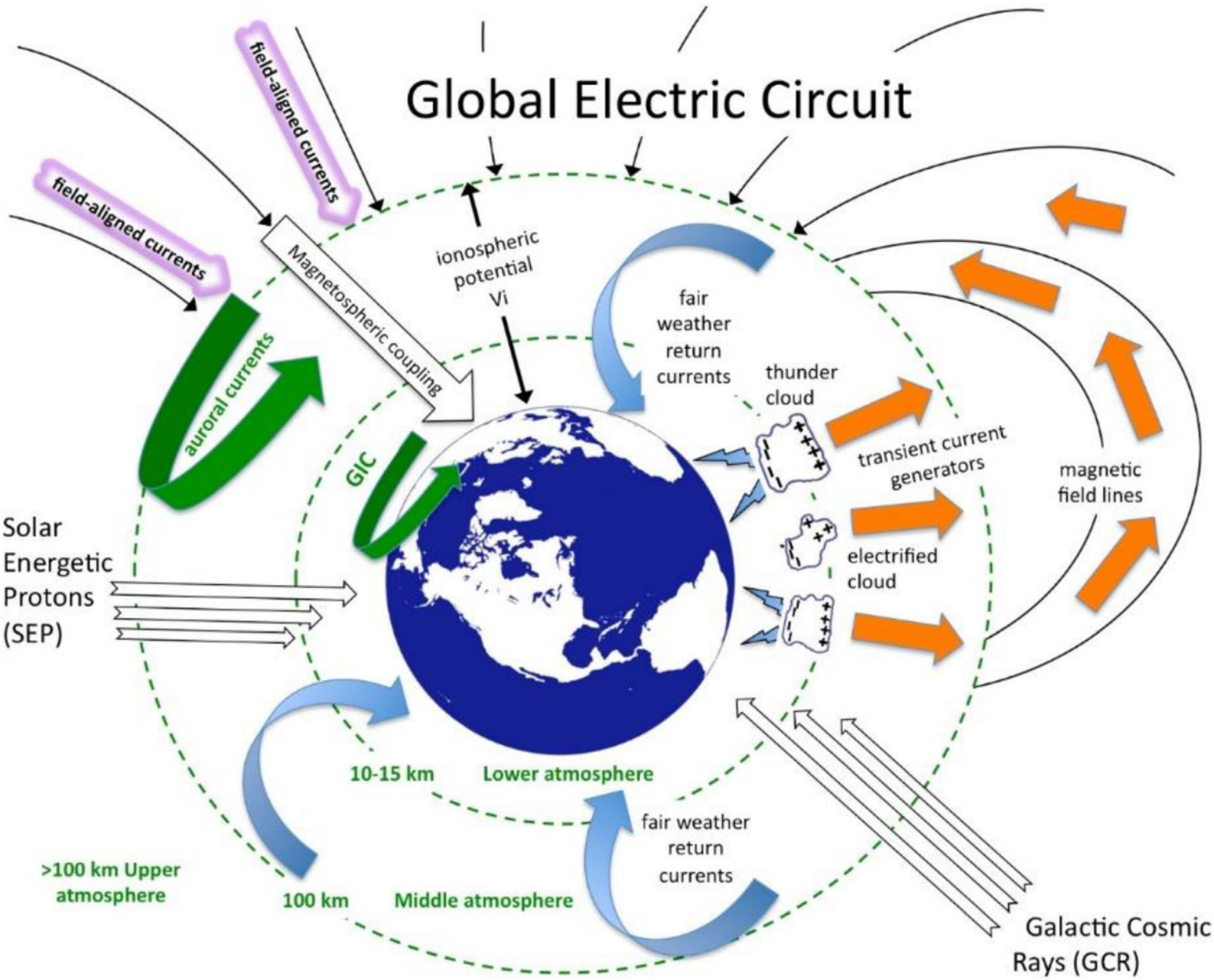
Cosmic and atmospheric phenomena that collectively drive the global electric circuit. Courtesy: National Science Foundation

Static electric fields are also pervasive throughout the Earth’s atmosphere as part of the global electric circuit that extends from lower ionospheric layers to the surface of the Earth (see Fig. 1 for an overview of the global electric circuit). In the lower atmosphere, a vertical potential difference exists, the potential gradient (PG), which is fuelled by a positively charged atmosphere and mobile electrical charges within the Earth system. This charge separation generates an electric field between the atmosphere and the Earth during fair weather conditions ranging between 100 and 300 V/m generating a direct current (DC) with a density around 2 pA/m^2^ (Israël 1971, 1973). These fields exist due to global thunderstorm activity hotspots that push positive charges towards fair weather regions and do so at a global scale (Haldoupis et al. 2017). Near local thunderstorms or in the presence of low clouds carrying or generating local charges (Harrison et al. 2017), however, this electric field becomes erratic with alternating positive and negative potential gradients that can exceed 10 kV/m (Williams and Mareev 2014). The PG is further influenced by local and regional factors, including vertically extending conducting objects (e.g., buildings and vegetation), natural ionizing radiation (e.g., radon decay), solar and auroral activity, the synoptic weather situation, desert dust storms or volcanic ash, and human-induced air pollution (e.g., Leblanc et al. 2008; Matthews et al. 2019; Kourtidis et al. 2020). The PG can show distinct daily variations that depend on the regular fluctuations of the global electric circuit, a variation commonly known as the Carnegie curve (see Harrison et al. 2013). The PG is also known to be altered by variations caused by local influences, e.g., aerosol particle pollution and radioactivity of the air (Reiter 1985). Seasonal variations have also been reported (Adlerman and Williams 1996), whereby the PG typically decreases during summer months.

Both electromagnetic and electrostatic fields are mostly, but not exclusively confined within the vertical atmospheric boundaries formed by the Earth’s surface and ionosphere (Volland 1995a, b). The Earth’s surface material is regarded to be a reasonably good conductor, and so is the lower ionosphere (60–130 km). Arguably arbitrary, these boundaries are considered to play a significant role in the presence and dynamics of the global circuit by providing a waveguide for the electromagnetic (EM) radiation (Rycroft et al. 2008). Although outside the scope of this review, it is important to mention that different EM frequencies across the spectrum will exhibit and experience different behaviours in the atmospheric medium and its complex and heterogeneous chemical composition within the equally diverse and changing boundaries (Volland, 1995a). Practically and measurably, this configuration is held responsible for generating a resonance cavity particularly suitable for the waveguide transmission of radio waves in the extremely low frequency (ELF, 3 Hz–3 kHz) and very low frequency (VLF, 3–30 kHz) range, as well as all forms of electromagnetic radiation (Volland 1995b). Remarkably, some distinct radiofrequency bands are naturally produced by lightning discharges across the globe (Volland 1995b; Price 2016). Natural waves of ultra-low frequency (ULF, 300 Hz – 3 kHz) can also enter the Earth’s atmosphere from the magnetosphere and ionosphere, from where they propagate along geomagnetic field lines as so-called geomagnetic pulsations or ionospheric Alfven resonances (Guglielmi and Pokhotelov 1996). Throughout the atmosphere, ELF electromagnetic waves called Schumann resonances (SR) (e.g., Price 2016) can be measured that result from global lightning discharges in the ground-ionosphere bounded waveguide. These weak waves have peaks at around 7.8, 14.3, and 20 Hz and can show some variations in frequency (± 0.2 Hz) and amplitude depending on the time of the day, the season, and, for instance, the modulation of the height of ionospheric layers due to solar activity.

The Earth’s atmosphere also has a number of local sources of electric variations that, in addition to contributing to local alterations of global patterns in atmospheric electricity, directly govern the local electric landscape and potentially the organisms living therein. These include profound impacts of local thunderstorm activity and in particular lightning strikes (e.g., Schaller et al. 2013), the production of ions through corona discharge (e.g., Matthews et al. 2010), radionuclides (e.g., Krivolutsky and Pokarzhevsky 1992), and the increasing use of electrical technology and devices (e.g., radio’s, portable communication devices) that contribute to shaping the local electric landscape.

## Atmospheric electricity and biological systems

### Electromagnetic fields

The extent to which geomagnetic and electromagnetic fields and waves affect biological organism has grown into an increasingly important field of study over the last century. The natural electrical, magnetic, and electromagnetic environment created by the existence of a conductive medium, current sources, charge separations, and ducts for wave propagation is admittedly complex. Beginning during the industrial revolution, humans increasingly generated artificial electric fields resulting from developments in industry and especially telecommunication technology, power lines from the electrical grid, transport, and a plethora of consumer electronics. This substantially enhanced the scale and complexity of the electromagnetic environment. In effect, whether naturally or technically generated, variable electrical currents are a substantial source of electromagnetic radiation in the atmosphere. For example, all manners of radio-communication, ranging from older radiolocation, radio-navigation, and portable telephones to the upcoming 5G wireless communication network work in the higher range of frequencies and are widely used in domestic, medical, and industrial appliances (Agiwal et al. 2016).

In biological systems, alternating currents (AC) range from a fraction of hertz to approximately 1000 Hz. Early studies focussed on biological effects of electromagnetic fields in the ELF range in relation to possible effects since ELF is measurable—albeit weak in comparison—in the activity of the human central nervous system (e.g., König et al. 1981). An increasing interest in higher radiofrequencies and microwaves subsequently developed due to the growing application in radio-communication and industry (Repacholi 1998). This interest persists as ELF is now nearly ubiquitous in both industrial and domestic environments (e.g., Bortkiewicz et al. 2006). ELF has also been considered to present potential health or therapeutic applications (König et al. 1981). While biological organisms have been naturally subjected to geomagnetic and electromagnetic fields over the course of the evolution of life on earth, scientific knowledge on the possible beneficial or deleterious effects of such fields remains sparse. Yet, evidence points to responses of biological systems, albeit inconsistent, to the action of electromagnetic fields and waves, including the current expansion of 5G wireless communication with potential adverse effects on DNA and membrane integrity, sperm function, and viability as well as immune and neuronal functioning (Marron et al. 1975; König et al. 1981; Liboff et al. 1984; Mikolajczyk 1990; Bortkiewicz et al. 2006; Valberg et al. 2006; Huss et al. 2007; Engels et al. 2014; Panagopoulos et al. 2015; Kocaman et al. 2018; Russell 2018; Saliev et al. 2019).

While mechanisms underlying the effects of both natural and artificial electromagnetic (EM) fields on biological systems can be expected to be the same, they are not necessarily easy to detect or describe over various levels of biological organization. For instance, effects at the molecular level can already be described with atomistic details, but at the level of cells or tissue require rather profound physical approximations and simplification (Cifra et al. 2020). To date, the cell membrane has been considered a major target of the electric field component of EM field (e.g., Azan et al. 2017), and much less attention has been paid to the direct effects of electric fields on proteins. However, intense electric fields at the nanosecond timescale have been shown to alter protein folding and structures (Marracino et al. 2019; Chafai et al. 2019). This may prove relevant given that proteins are biological nanomachines that execute the vast majority of life processes, so any direct action of EM fields on proteins might have substantial downstream effects.

The fact that variations in atmospheric electric fields have been observed to be biologically relevant to organisms and processes in the natural environment has also encouraged research aimed at disentangling the links between large natural and anthropogenic fluctuations in atmospheric electricity and human well-being. Interactions of atmospheric electricity with human health can be by characterizing anomalous electric environments where unusual biophysical responses in humans become visible (Cannon 1929), although it is difficult to define the personal limits of exposure to natural electric variations. Various atmospheric physical properties have been proposed to be potentially relevant. Although natural electromagnetic fields are generally weak, large-scale variations in various atmospheric phenomena (e.g., radiation, electro-magnetic fields, lunisolar gravitational forces) have been observed to affect cardiovascular systems and biological rhythms (Sollberget 1963; Halberg 1963; Palmer et al. 2006), suggesting local and planetary electrical phenomena have the potential to influence—at least part of—the human population.

### Electromagnetic resonances

Lightning discharges generate electromagnetic resonances excited within the Earth-ionosphere waveguide across the globe, the so-called Schumann resonances (SR; Schumann 1952; Price 2016). Lightning events produce signals that are very weak (~ 300 μV m^−1^ and below 100 Hz) and typically have a low spatial attenuation rate (0.5 dB/Mm), allowing electromagnetic waves from an individual discharge to propagate several times around the globe before it eventually decays (Bliokh et al. 1980). In this physical context, the Earth-ionosphere waveguide behaves like a resonator at extremely low frequencies. This waveguide behaviour results in the amplification of spectral signals from lightning at resonance frequencies due to interference of EM waves propagating in opposite directions around the globe. It is believed that SR has existed throughout the course of Earth history after the formation of the atmosphere (Kasting and Siefert 2002), suggesting SR could be a physical quantity that, much like light, sound and gravity, could have a constituted part of the adaptive landscape in the early evolution of life (e.g., Price et al. 2020). SR occur in the ELF range, with resonant frequencies around 8 Hz, 14 Hz, 20 Hz, 26 Hz, etc. Many living organisms in nature also show electrical activity in the ELF range. From zooplankton to sharks in oceans to the human brain, all show spectral activity between 4 and 40 Hz (Bullock 2002; Freund et al. 2002). For example, the normal brain activity in humans at rest is around 10 Hz (Nunez et al. 1978), between the first two resonant frequencies of the SR. The question remains whether there is a connection between naturally produced SR and organisms and whether organisms have evolved the ability to sense and process the information that is hidden in its weak electric fields (Cherry 2003). Research has shown that entire organisms can be influenced by weak SR fields around 10 Hz (Wever 1973). As early as the 1960s, studies on circadian rhythms have shown that weak 10 Hz SR fields can influence the daily activity cycle of humans, birds, and fruit flies (Wever 1973; Engelmann et al. 1996). Recently, cardiac muscle cells were observed to be influenced by weak magnetic fields in the SR frequency range (Elhalel et al. 2019), which appeared dependent on the frequency (most pronounced at 7.8 Hz) rather than amplitude of the induced field. These studies collectively suggest that very weak alternating magnetic fields can indeed potentially influence biological processes and human health, yet a physical understanding of these findings is still absent (Price et al. 2020; Fdez-Arroyabe et al. 2020).

### Static atmospheric electric fields

In the fair-weather regions across the globe, a static atmospheric electric field of the order of amplitudes ca. + 100 to + 300 V/m occurs as a consequence of the global atmospheric electrical circuit. Directed downwards if considered as a vector electric field, this atmospheric potential gradient (PG) undergoes various variations that can be regular (e.g., daily and seasonal) or irregular (locally driven) (Rycroft et al. 2008). The relevance of static atmospheric electric fields for biology has only recently been considered, with a particular focus on the relationship between insect pollinators and plants (e.g., Clarke et al. 2013). It has been found that flowers are surrounded by an electric field that results from a combination of a plants’ placement in the atmospheric PG and electrochemical fluxes through their vascular system and the ground (Maw 1962; Volkov and Shtessel 2018). Hence, a relative negative potential can be observed between flowers and the atmosphere. Several lines of research have assessed this electrostatic linkage and demonstrated empirically that electrostatic forces play a role in the transfer of pollen from flower to pollinator (Armbruster 2001; Corbet and Huang 2014; Clarke et al. 2017). Furthermore, evidence has emerged that bees can detect and use the floral electric fields to associate reward (nectar or pollen) with flowers (Clarke et al. 2013), providing the first documentation of electroreception in air as a resistive medium (Clarke et al. 2013; Greggers et al. 2013). The atmospheric PG also bears direct importance for other arthropods. It was recently shown that spiders can use the electric field in fair weather to balloon upwards in attempts to disperse over longer distances, in which the electric force acts on casted thin strands of silk allowing them to take flight (Morley and Robert 2018). The atmospheric PG has also been observed to extend below the Earth’s surface layers, in which a charge separation between relatively negative soils and sediments and the relatively positive overlying atmosphere results in the movement of respiratory ions and altered bacterial metabolism in subsurface environments (Hunting et al. 2019). The resulting alteration in microbial communities and their metabolic activities likely has wider implications as they serve as a food source for higher trophic levels (Zhai et al. 2018) and are essential for ecosystem processes like decomposition (Hunting et al. 2017). Altogether, these studies indicate that static atmospheric electric fields and their variability are tied to various biological processes, warranting further investigations assessing its significance within an ever-changing and often elusive electrostatic landscape. This encourages efforts to better understand the structure and dynamics of static electric fields at the spatial and temporal scales that are relevant for a potentially wide array of organisms that may use the dynamic electric landscape above, near, and directly below the surface of the Earth.

### Lightning

Lightning is a ubiquitous phenomenon on Earth with around 50 lightning strikes per second (Christian et al. 2003). When lightning hits the Earth’s surface, electric current flows through paths of higher conductivity or moisture content (e.g., plants and soil). The energy contained within lightning strikes causes rapid heating of Earth’s surface environment, whereby temperatures may exceed 2500 K (Pasek and Block 2009). Aside from direct effects on biology, it is thus conceivable that these electric currents and associated energy inputs can bear relevance for biology.

**Fig. 2:**
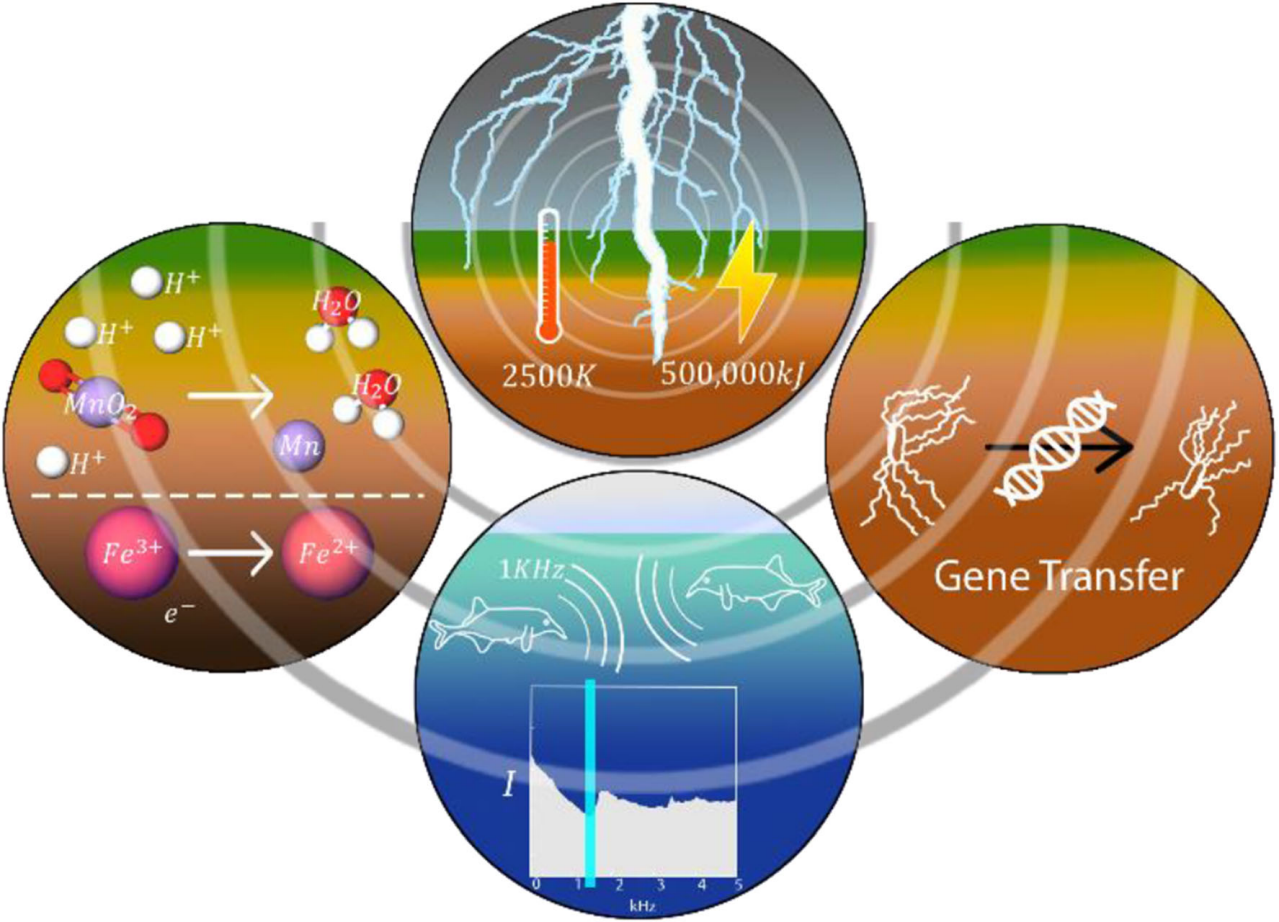
Overview of geochemical and biological phenomena that are directly or indirectly affected by lightning. Top: lightning strikes generate 500,000 kJ of energy and heat the ground to 2500 K. Left: lightning reduces soil elements including manganese and iron, increasing their mobility (Schaller et al. 2013). Bottom: Mormyrid fish communicate using frequencies (vertical band on graph) where noise from lightning is lowest (Hopkins 1980). Right: current from lightning mediates gene transfer in soil (Demanèche et al. 2001)

Lightning strikes have long been known to affect biological systems by directly causing injury or death, notably in cattle, humans, and trees (Bernstein 1973; Kautz et al. 2011). While direct effects of lightning on biology are generally obvious, less obvious indirect effects have also been observed (Fig. 2). Lightning can generate electrical noise influencing electrical communication in freshwater electric fish (Hopkins 1973, 1980). Indeed, *Gabon mormyrid* fish appear to use electrical organ discharge frequencies around 1000 Hz; the low noise bandwidth window, where there is no transmission, allows enhanced propagation of EM energy (Hopkins 1980, Arnason et al. 2002). In addition, lightning and resulting soil currents have been identified as a driver of the transfer of genetic material between different bacterial species (Demanèche et al. 2001). More recently, lightning has also been associated with chemical alterations in the Earth’s surface. This is relevant for organisms living in this environment; subsurface electrochemistry and in particular microorganisms are known to be strongly interdependent (Newman and Banfield 2002; Naudet and Revil 2005; Hunting et al. 2012, 2015; Hunting and Kampfraath 2013). Specifically, lightning has been observed to reduce phosphorus, an important nutrient for microorganisms and phototrophic organisms (algae, plants) in terrestrial and aquatic environments (Pasek and Block 2009). Lightning has also been observed to enhance mobilization of metals in both soils and aquatic sediments, potentially enhancing availability of essential metals or their toxicity to organisms (Schaller et al. 2013). These studies provide important clues on the significant and persisting effects of lightning on the abiotic and biotic environment, upending the steadfast view that lightning is a local and transient phenomenon.

### Ions and aerosols

The atmosphere contains a wide variety of ions that can differ significantly in size and charge. These electro-active ions have a tendency to attach to aerosols and originate from both natural (cosmic rays, radioactivity, splashing water, dust storms) and anthropogenic sources (high voltage infrastructure and exhaust fumes from traffic). For instance, a substantial number of corona ions are produced by high-voltage power lines when the voltage is high enough to cause corona breakdown around the cable (Matthews et al. 2012; Jayaratne et al. 2015). If there is a predominance of one polarity of ion, such as near DC power lines and some AC power lines, this can lead to the enhancement of aerosol charge. For AC power lines, the amount of corona has been shown to be affected by local meteorology and time of day (Matthews et al. 2012).

Anthropogenic infrastructures (e.g., high-voltage transmission lines) are generally considered as the main source of this kind of ions (e.g., Matthews et al. 2010). The vast majority of studies hitherto focused on how ions influence microorganisms and human health using model organisms (e.g., mice; e.g., Krueger et al. 1963; Berger et al. 1976; Brun et al. 2018) and effects of increased air ion concentrations on biological systems have been noted (Harrison and Carslaw 2003). For instance, natural ionization of the air has long been known to be bactericidal and to disrupt levels of the neurohormone serotonin (Krueger and Smith 1958) and reduce lifespan in mice (Krueger and Reed 1976; Kellogg III and Yost 1983). The charging of aerosol has also been speculated to lead to an enhanced deposition due to electrostatic effects, potentially increasing deposition of harmful material on the skin (Fews et al. 1999a) or lung tissue through inhalation (Fews et al. 1999b). This has been offered as an explanation for increased rates of childhood leukaemia near high-voltage power lines in some studies (Tynes and Haldorsen 1997; Draper et al. 2005). Enhanced deposition within the lung has been demonstrated within mechanical models of the lung (Cohen et al. 1996) and with multiple charged particles larger than 300 nm in adult human volunteers (Melandri et al. 1983). Yet, air-borne particles measured near to HV power lines represent a relatively low charge enhancement compared with those which have so far demonstrated an effect (Buckley et al. 2008; Matthews et al. 2015; Usmani et al. 2020). It is important to note, however, that effects of ions on biological systems can be caused by electrodynamic, electrostatic, or electrochemical (e.g., ozone production) mechanisms (Fletcher et al. 2007), suggesting a need to control for confounding variables.

### Radionuclides

Unstable atoms in the atmosphere, radionuclides, also contribute to the complexity of variations in local atmospheric electricity through ionizing radiation. Most atmospheric radionuclide species originate from the transfer of radioactive material from the Earth surface (e.g., radon) or from extra-planetary ionizing radiation (e.g., cosmogenic beryllium). Among the natural radionuclides, radon and its decay products are considered major contributors to health risk to living organisms, with radon being the second leading cause of lung cancer after tobacco smoke (Sethi et al. 2012). It has been demonstrated in many studies that radionuclides derived from nuclear weapons testing and nuclear accidents can influence the electrical properties of the atmosphere (Israelsson and Knudsen 1986; Tuomi 1988; Yamauchi et al. 2012). Radionuclides can therewith have further direct and indirect effects on organisms: exposure can cause direct effects such as increases in illness or death and result in genotoxic effects such as single- and double-strand deoxyribonucleic acid (DNA) breaks or DNA alterations (Ward 1995), chromosomal aberrations (Geraskin et al. 2003), or morphological abnormalities (Hiyama et al. 2012). Indirect effects of exposure can include suppression of radiosensitive species, disruption of trophic relations, a loss of immunity, and occurrence of novel diseases (Geraskin 2016). For instance, changes in community composition of plants (Suvorova et al. 1993) and soil fauna (Krivolutsky and Pokarzhevsky 1992) have been observed in areas affected by the 1986 Chernobyl nuclear power plant accident.

## Implications and future challenges

### Methodological challenges

Progress in our understanding of the electric landscape and its biotic constituents is hindered by technical challenges and limitations. The electrical environment is described by interdependent physical parameters (e.g., current, conductivity, electric field, charge location, number, and mobility). Measurement techniques exist for these electrical parameters (Harrison and Ingram 2005; Harrison 1997; Aplin and Harrison 2000; Chubb 2014), but they vary across large spatial and temporal scales, and range across many orders of magnitude (e.g., 10^−15^ to 10^3^ A currents) for which logarithmic high dynamic range sensors can be required (Marlton et al. 2013). Therefore, sensors are used in arrays, which impose practical constraints such as size and ease of deployment. Likewise, sensors are designed to have the appropriate bandwidth and range to meet the specific scientific questions considered. It is often not feasible to meet all these requirements and, as a result, a variety of different sensors are often needed. Another challenge is to partition the significance of all atmospheric (electric) phenomena that operate simultaneously to directly and indirectly affect the living environment. Therefore, simultaneous measurements of several parameters are often needed to be able to disentangle and partition multiple confounding factors. Miniaturization and integration of several different sensors in a robust and easily deployable measurement package would offer the opportunity to gather more complete and continuous data of these drivers simultaneously and identify key parameters in the interaction between atmospheric electricity and biological systems.

In practise, methodological challenges can be met when experimental protocols ideally necessitate strict controlling and manipulating of the electric fields involved. Substantial difficulties are recognized to arise when a wide range of frequencies have to be shielded from the experimental subject in laboratory situations. Experimental manipulations, including important sham controls, set-up symmetry, stimulus isolation, and other conventional quantification of dose-responses, are not trivial and often onerous. The exploration of the entire parameter space, from DC to GHz frequencies, is desirable yet challenging logistically and financially. One additional challenge stems from the need to document the wave forms and incident magnitudes of exposures and reproduce them in controlled laboratory conditions, in the presence of other physical and biogenic variables. Hence, to date, difficulties remain in designing meaningful and interpretable empirical investigations involving biological systems and their responses to EM fields, which in turn, can be expressed at multiple levels of biological complexity, e.g., behaviour, physiological, molecular, and atomic. It must be recognized that the reproducibility of methodologies, and hence repeatability of experiments, has been an issue in the vast majority of studies published to date, casting uncertainty on our capacity to formulate a solid phenomenology on the effects of atmospheric electricity on biological organisms, including humans.

In studies focussing on human health, the role of atmospheric electricity remains uncertain due to the many external factors, which partially or entirely control exogenous and endogenous biological rhythms. Appropriate control and manipulation of circadian rhythms is thus key for successful experiments (Halberg and Panofsky 1961). The large number of confounding variables in atmospheric parameters, geographic distributions, and lifestyle variability makes this field notoriously challenging. To facilitate progress, a key challenge is the development of Biometeorological Data Infrastructures (Fdez-Arroyabe et al. 2018). These infrastructures can be based on monitoring people and animals in order to collect data and define the vulnerability of individual organisms as well as populations to acclimatize and adapt to normal variability and extreme changes of specific atmospheric parameters. The development of biometeorological data infrastructures based on empirical measurements would be the first step in advancing our understanding on human well-being in relation to its atmospheric electrical environment and ultimately allow for developing tailored early warning systems that could mitigate risks for individuals and populations.

### From electrons to ecosystems

The scales at which atmospheric electric phenomena act range from particles to global circuits. How these phenomena interact with different levels of biological organization, which themselves vary spatially and temporally, constitutes a daunting challenge. The electrical landscape of any biome will be a product of the dynamic interplay between abiotic sources (e.g., atmospheric potential gradient) and perturbations by living organisms. Ultimately, for nearly all environments on Earth, abiotic and biotic components will be both sources and sinks, as well as modifiers, of electric fields and ions that interact in intrinsically linked and reciprocal ways. However, the vast ranges in spatial scale and magnitude over which these interactions occur make accurate measurements and comprehensive modelling of the dynamics of this system and its constitutive components a challenging and worthwhile task.

Despite the various interdependent electric and electromagnetic phenomena, not all are expected to be sufficiently strong enough to exert an observable effect on biology, and effects can be expected to differ across various levels of biological organization (e.g., molecules, cells, and organisms: see Cifra et al. 2020 for review). Molecular dynamic simulations (Průša and Cifra 2019; Valle et al. 2019) and further modelling are currently used to identify under what conditions atmospheric electric and electromagnetic fields can modify functions of molecules, an approach that also enables a prediction of the effects on other molecules and organelles (e.g., Tuszyński et al. 2005). While we thereby begin to understand the effects of atmospheric electricity on molecular level processes, a major challenge remains to upscale this analysis to cell and tissue levels, or beyond. Disentangling molecular dynamics at atomic precision could provide a valuable bottom up approach that can inform higher scales of application and complexity in modelling (Apollonio et al. 2013).

**Fig. 3:**
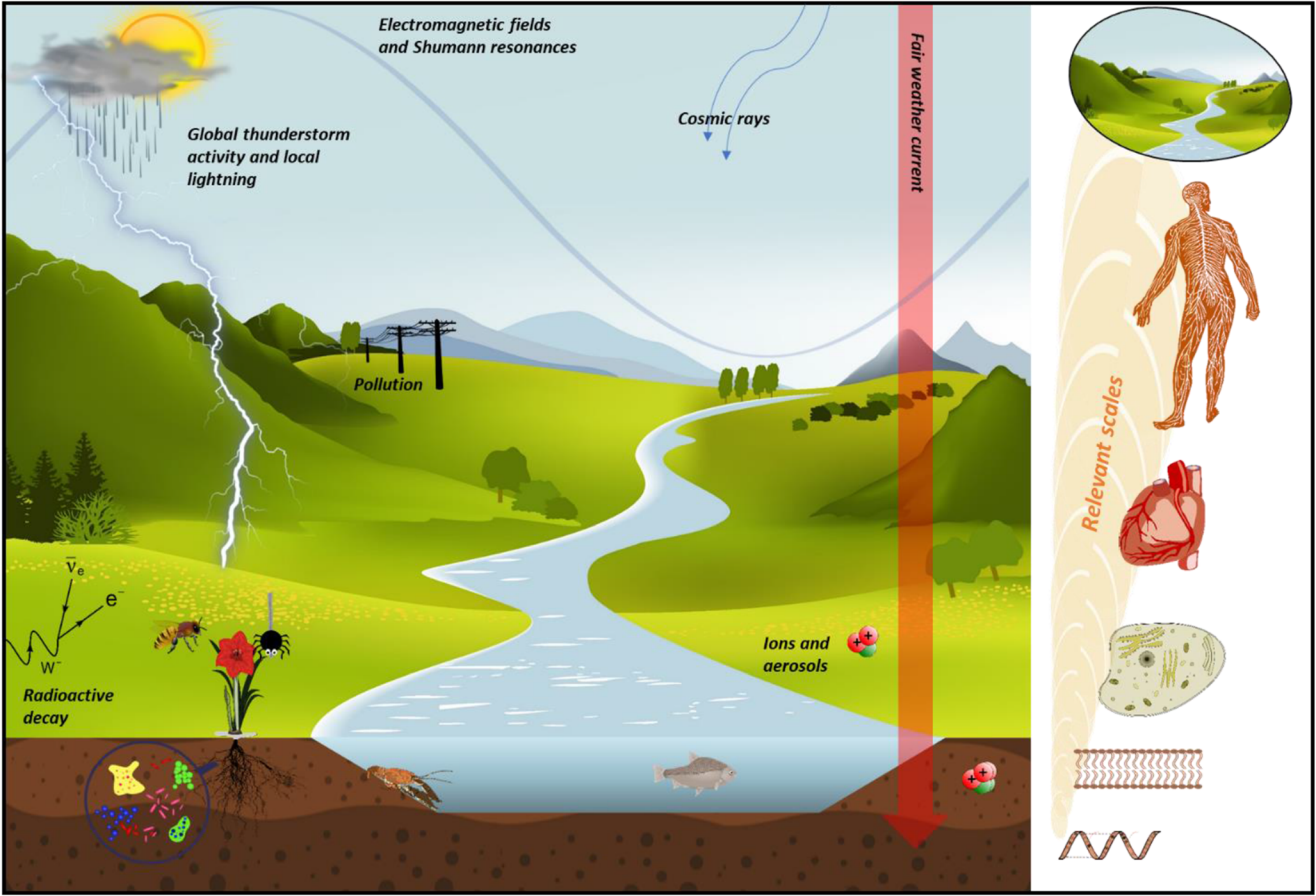
Conceptual diagram illustrating various atmospheric electric phenomena that have demonstrated links with various levels of biological organization. While a plethora of studies examined effects on a molecular level and we begin to increase our understanding of higher levels of molecular and cellular organization, a consideration of a wide range of spatial and scales in atmospheric electricity is required to identify links across all levels of biological organization and how they propagate across ecosystems in time and space commensurate with the life cycles of terrestrial organisms

Although challenging, consideration of a wide range of spatial scales in atmospheric electricity is required to identify links across all levels of biological organization (see Fig. 3 for an overview of electrical phenomena tied to different levels of biological organization). For example, the exchange of a relatively small number of electrons on the surface of an insect’s mechanosensory hair could potentially lead to drastic differences in its sensitivity to electric field in a behavioural context stimuli (Sutton et al. 2016). Conversely, on a larger scale, the shielding and distortion effects imparted by trees on the atmospheric potential gradient can effectively nullify or transform the local electric field strength experienced by organisms in their immediate vicinity (Arnold et al. 1965; Williams et al. 2005; Clarke et al. 2017). Likewise, relationships between plants and the atmospheric PG are likely to be species-dependent, owing to species-specific morphology and electrophysiological characteristics. Furthermore, at an even greater scale, the burning of organic matter, as naturally occurs in forest fires, has been suggested as a significant source of negative ions, resulting in anomalous lightning strikes over large areas (Vonnegut et al. 1995). Adding further complexity, many of these interactions transcend multiple tiers of scale, with the largest scale atmospheric electric fields having a marked influence on some of the smallest levels of biological organization. For instance, it has been noted that both local and universal periodic variations in atmospheric electricity can influence the subsurface electrochemistry of soils and water-bodies (Hunting et al. 2019). These changes in electrochemical gradients alter the metabolic activity of microorganisms (Hunting et al. 2019) and could potentially influence the movement of electrotactic organisms (Bespalov et al. 1996; Chrisman et al. 2016). This could further extend to large-scale variations in space weather that are known to influence surface atmospheric electricity (Harrison et al. 2013).

While the basics of electrostatics (Faraday 1839) and electrodynamics (Maxwell 1865) have long been described, the complexity of the biotic environment constitutes a challenge by itself. Through both its physical structural and material diversity, and the possibly countless electrical interactions within, biological material renders the application of said fundamental principles extremely difficult in a biologically relevant setting. In effect, a better identification of the suite of interactions between abiotic and biotic electric fields is much needed to warrant progress in this field. This endeavour, in tandem with measurements and modelling of the electric fields present in complex organic environments, should begin to allow for characterization of the living electrical landscape, and the dynamics therein. Identifying the aspects of the natural electric atmospheric landscape in conjunction with the anthropogenic electric landscape will ultimately allow for establishing a complete picture amenable to experimentation and the gathering of empirical evidence.

### Human activities and atmospheric electricity

An increased recognition of a coupling between the electric landscape and biological systems also calls for investigating to what extent this coupling is vulnerable to anthropogenic influences. Various sources of anthropogenic pollution have been identified, ranging from smoke to power lines, which vary in their degree in which they affect the local electric landscape. For instance, smoke and aerosols are known to affect atmospheric electricity (Sheftel and Chernyshev 1994; Kamra and Deshpande 1995; Maricq 2006), and although the number of particles from traffic decays quickly (~ 10 m) (Lee et al. 2012), they can exceed particle numbers near power lines (Maricq 2006; Jayaratne et al. 2015). More pervasive are the effect of electrical wires and power lines. The 50 or 60 Hz “mains hum” can even be detected in aquatic habitats (Peters and Bretschneider 1972), and electrical pollution by high-voltage power lines is a wide spread factor affecting local variations in AE (Maruvada 2011) that can be measured hundreds of meters away from power lines (Matthews et al. 2010, 2012).

Whether sources of anthropogenic pollution affect the electric landscape sufficiently enough to influence biology remains a central issue and studies are often ambiguous. Power lines have been observed to trigger behavioural responses in insects and planarians (Jackson et al. 2011; Petri et al. 2017; Schmiedchen et al. 2018), but no physiological mechanism underlying these observations has been detected so far. It has also been proposed that resulting fluctuations in E-fields can be disruptive to circadian rhythms (Henshaw et al. 2008), and power frequency fields have resulted in melatonin disruption in rats (e.g., Wilson et al. 1981; Wilson et al. 1986; Reiter et al. 1988; Grota et al. 1994). In addition to AC and DC fields, power lines can shed ions, thereby providing a secondary and indirect source of electrical pollution that potentially alters local direct current and ion transport, adding further complexity. The myriad potential perturbations caused by human activities may therefore—in concert—interfere with linkages between atmospheric electricity and biological systems in ways that remain largely unexplored.

## Concluding statement

Collectively, the research reviewed in this article serves to document and highlight the links between atmospheric electricity and biological systems. The evidence presented illustrates the multiple facets of current research while shedding light on gaps that warrant investigation. One key emerging perspective is the expectation that variations in atmospheric electricity affect various biological systems across multiple ecosystem boundaries. It is also becoming apparent that in addition to directly influencing biology, atmospheric electricity can have various indirect links to organisms and biological processes. Technical and methodological challenges create a number of pitfalls that prevent the gathering of conclusive evidence and warrant the development of interdisciplinary research that seeks the integration and harmonization of research disciplines such as atmospheric physics, biometeorology, behavioural and sensory biology, ecology and ecophysiology, and medical and environmental sciences. While many examples show the interactions of atmospheric electrical phenomena at multiple organizational scales (e.g., effects on molecules, cells, and organisms), it becomes progressively more important to consider wider spatial and temporal scales. At stake is a deeper understanding of how and why diverse interactions can propagate across ecosystems in time and space commensurate with the life cycles of terrestrial organisms.
